# The Mental “Weight” of Discrimination: The Relationship between Perceived Interpersonal Weight Discrimination and Suicidality in the United States

**DOI:** 10.1177/00221465231200634

**Published:** 2023-09-30

**Authors:** Carlyn E. Graham, Michelle L. Frisco

**Affiliations:** 1 The Pennsylvania State University, University Park, PA, USA

**Keywords:** adults, suicide attempts, suicide ideation, United States, weight discrimination

## Abstract

Extant research has investigated the relationship between body weight and suicidality because obesity is highly stigmatized, leading to social marginalization and discrimination, yet has produced mixed results. Scholars have speculated that factors associated with body weight, such as weight discrimination, may better predict suicidality than body weight itself. We consider this possibility among a sample of 12,057 adult participants ages 33 to 43 in Wave V of the National Longitudinal Study of Adolescent to Adult Health through investigation of the relationships between weight discrimination and two dimensions of suicidality—suicide ideation and attempts. We also examine gender as a moderator of these relationships. We find that weight discrimination is positively associated with both suicide ideation and attempts, and this relationship is similar among men and women. Our findings underscore the need to address issues of weight discrimination in our society to better promote mental well-being.

Suicide is one of the top five leading causes of death among adults between the ages of 25 and 44 (Centers for Disease Control and Prevention 2023). Among men in this age group, the suicide rate is second only to the rate among men ages 75 and older (National Institute of Mental Health 2022). Among women in this age group, the suicide rate (7.2 per 100,000) is nearly identical to the age group with the highest suicide rate—women ages 45 to 64 (7.9 per 100,000; National Institute of Mental Health 2022). Men and women ages 25 to 44 also have the second highest prevalence of suicidal thoughts and attempts (National Institute of Mental Health 2022).

Although not equivalent to suicide mortality, suicide ideation and attempts are among the most salient risk factors for it (Klonsky, May, and Saffer 2016). A previous suicide attempt is the greatest predictor of future death by suicide (Irigoyen et al. 2019). Given that suicide is a leading preventable cause of premature mortality among younger adults, identifying factors that lead them to consider and attempt suicide is needed.

Previous research has examined the relationship between body weight and suicidal thoughts and behaviors because obesity is highly stigmatized (Jackson 2016; Pearl 2018; Puhl and Heuer 2009), leading to discrimination (Carr and Friedman 2005; Spahlholz et al. 2016), social rejection (Pont et al. 2017; Puhl and Latner 2007), and social marginalization (Apolloni, Marathe, and Pan 2011; Strauss and Pollack 2003). Studies of body weight and suicidality are often framed within the interpersonal theory of suicide (IPTS), which argues that individuals in higher weight bodies may have an increased risk for suicidal thoughts and behaviors because of low self-worth, self-hate, thwarted belongingness (i.e., an absence of reciprocal and caring relationships; Zuromski et al. 2017), and perceptions that they are a burden on others or society (Dutton et al. 2013; Zuromski et al. 2017).

However, the evidence about how body weight and suicidality are related is inconsistent (for a review, see Perera et al. 2016). Among U.S. adults, in particular, some studies point to a positive association between obesity and suicidal ideation (Dong et al. 2006; Dutton et al. 2013; Zuromski et al. 2017), whereas others show a null association (Graham and Frisco 2022; Zhao et al. 2012) or even a negative association among men (Carpenter et al. 2000; Zhang 2006).

Given these inconsistent findings, researchers have speculated that suicidality may be more closely linked with factors such as weight perceptions (Haynes et al. 2019), stigma (Haynes et al. 2019), and discrimination (Graham and Frisco 2022; Hunger, Dodd, and Smith 2020). This is because overweight perceptions, regardless of weight status, may lead to the fear of being devalued, stigmatized, or discriminated against (Hunger et al. 2015), all of which has been theorized to foster feelings of perceived burdensomeness and thwarted belongingness (Douglas, Kwan, and Gordon 2021; Hunger et al. 2020). To date, studies have shown that factors such as overweight perceptions (for a review, see Haynes et al. 2019), weight-based teasing (Eisenberg, Neumark-Sztainer, and Story 2003), body-related unfair treatment (i.e., body shape, size, or physical appearance; Sutin et al. 2018), and weight stigma (Douglas et al. 2021) are positively associated with suicidality, net of body weight, in studies of adolescents (Eisenberg et al. 2003; Haynes et al. 2019; Sutin et al. 2018) and a small sample of undergraduate psychology students at a midsize Midwestern U.S. university (Douglas et al. 2021).

To our knowledge, only one study has examined the relationship between weight discrimination and suicide ideation among U.S. adults. Hunger et al. (2020) found a positive association in analysis of data from two small samples of predominantly White, highly educated adults who participated in online surveys. Due to the limitations of their sample size, these scholars noted that their results should be considered preliminary until replicated in a better powered study.

In this study, we heed this call in analysis of data from Wave V of the National Longitudinal Study of Adolescent to Adult Health (Add Health). Adults in the age range of Wave V participants (33–43) have the highest reported prevalence of weight discrimination among adults between the ages of 25 to 74 (Puhl, Andreyeva, and Brownell 2008). This makes them an important focal group for research on weight discrimination and suicidality. We produce nationally representative estimates of how perceived interpersonal weight discrimination (hereafter, “weight discrimination”) is related to suicide ideation among 33- to 43-year-olds, net of body weight. We also investigate the relationship between weight discrimination and suicide attempts. Examining this second dimension of suicidality is a critical contribution to the literature because a history of suicide attempts is the most robust predictor of future suicide ideation, attempts, and mortality (Parra-Uribe et al. 2017). Moreover, suicide ideation and suicide attempts are not synonymous and should not be treated as such, given that most individuals with suicide ideation do not attempt suicide (Klonsky et al. 2016). Our final analytic goal is investigating whether gender moderates the relationship between weight discrimination and both study outcomes. This is an important line of investigation because women are disproportionately more likely than men to experience weight stigma and discrimination (Pearl et al. 2018; Puhl et al. 2008; Spahlholz et al. 2016) and to internalize weight bias (Boswell and White 2015; Himmelstein, Puhl, and Quinn 2017; Puhl, Himmelstein, and Quinn 2018).

## Background

### Body Weight, Weight Stigma and Discrimination, and Mental Health

In the United States, female slenderness and male muscularity signify willpower, restraint, moderation, and self-control (Saguy 2013). Female slenderness and male muscularity are also badges of high morality and status due to the societal perception that individuals with these body types are successfully managing their health (Gutin 2021). By contrast, obesity is regarded as health-harming and undesirable (Gutin 2021; Ringel and Ditto 2019). The U.S. “personal responsibility framework” blames individual choices about controllable lifestyle factors such as diet and exercise for obesity (Papadopoulos and Brennan 2015; Ringel and Ditto 2019; Saguy 2013). This leads to societal perceptions that larger bodies are the result of poor impulse control, lack of self-discipline or willpower, laziness, gluttony, greed, and self-indulgence (Carels et al. 2013; Gutin 2021; Ringel and Ditto 2019; Saguy 2013). Consequently, individuals in larger bodies face moral condemnation for failing to manage their weight and health (Ringel and Ditto 2019).

The moralization of obesity and negative perceptions about it leads to weight-based stigma and discrimination against individuals in larger bodies (Jackson 2016; Klaczynski, Goold, and Mudry 2004; Major, Eliezer, and Rieck 2012; Ringel and Ditto 2019). Discrimination is consistent with the concept of *enacted stigma* that denotes tangible actions (either interpersonal or structural) of unjust mistreatment toward stigmatized individuals based on negative stereotypes and preconceptions (Major and Schmader 2018). This is a primary reason that individuals in larger bodies are treated with less respect than others, receive poorer service than other people at restaurants and stores, are threatened and harassed, and are called names more often than leaner peers (Carr and Friedman 2005). Obesity is also associated with structural discrimination within settings such as the labor market, education, and health care (Carr and Friedman 2005; Jackson 2016; Pearl et al. 2018; Tomiyama et al. 2018).

Unsurprisingly, findings from a growing body of literature indicate that exposure to weight stigma or discrimination can increase the risk of adverse mental health outcomes such as experiencing a major depressive episode, depressive symptoms, psychological distress, stress, and/or anxiety (i.e., Emmer, Bosnjak, and Mata 2020; Hatzenbuehler, Keyes, and Hasin 2009; Pearl et al. 2018; Robinson, Sutin, and Daly 2017; Sutin et al. 2016). However, less attention has been given to the relationship between weight stigma or discrimination and suicidal thoughts and behaviors. This is a noteworthy gap because although mental health outcomes such as depression and anxiety are associated with suicidality (Fehling and Selby 2021), they are not prerequisites (Druss and Pincus 2000), especially in the case of suicidal behaviors (Fehling and Selby 2021; Klonsky et al. 2016). Moreover, the majority of individuals with mental health issues such as depression do not experience suicidal thoughts or attempt suicide (Pompili 2019). Thus, it is important to expand the existing literature to determine if weight discrimination is a risk factor for suicidality independent of other adverse mental health outcomes (Sutin et al. 2018).

### Theoretical Perspective on Weight Discrimination and Suicidality

The existing studies of how weight stigma and weight discrimination are related to suicidality among adults are motivated by the interpersonal theory of suicide (IPTS). As noted previously, this theory argues that individuals that experience weight-related stigma or discrimination are at risk for suicidal thoughts and behaviors because of thwarted belongingness and perceptions of being a burden (Douglas et al. 2021; Hunger et al. 2020). When tested empirically, these studies suggest that perceived burdensomeness, not thwarted belongingness, partially mediates the associations between weight discrimination and suicide ideation (Hunger et al. 2020) and between weight stigma and suicide risk (Douglas et al. 2021). As such, these studies and the IPTS framework have been helpful in identifying the psychological processes that lead individuals who experience weight discrimination and weight stigma to exhibit suicidality.

Yet it is also important to recognize that there are external, structural forces at play that lead to these psychological processes and the potential consequences of weight discrimination for suicidality. Neo-Durkheimian paradigms, particularly the reconceptualization of Durkheim’s theory of suicide as it relates to stigma and shame, are helpful in this front. To elaborate, failure to meet expectations, either individual or societal, contributes to feelings of shame and anomie (Abrutyn and Mueller 2014). In turn, shame induced by failure to meet expectations leads to a risk of suicidality (Kalafat and Lester 2000). More explicitly, shame encompasses feelings of rejection from social bonds (Johnson 2020; Mokros 1995) and threats to social identities that are rooted in expectations set through pivotal relationships to individual people, groups, social statuses, and/or collective society (Mueller et al. 2021). Suicide (or suicidality) ends the pain associated with unbearable “self-ridicule” and internalized shame due to violated expectations and norms (Mokros 1995).

Abrutyn and Mueller (2014) argue that stigmatized identities are the result of violated cultural norms. They propose that the more salient that an identity is to an individual and the more stigmatized it is by a culture, the more a person will experience “engulfed shame,” increasing their likelihood for suicidality. Weight stigma embodies social rejection and devaluation due to failure to adhere to social norms about ideal body size (Tomiyama et al. 2018). Weight stigma is an especially pernicious type of stigma because weight is viewed as controllable in U.S. society (Major et al. 2012, 2018; Major and Schmader 2018; Schmitt et al. 2014). As such, individuals who experience weight stigma are more likely to perceive it as legitimate and internalize it than individuals who experience stigmas due to statuses and identities that are perceived as uncontrollable (i.e., race or ethnicity; Major et al. 2012; Schmitt et al. 2014).

Individuals facing weight stigma also face greater stigmatization than individuals facing other types of stigma (Major et al. 2018; Major and Schmader 2018) because they are sanctioned by society as “choosing” to be stigmatized (Major et al. 2018). This lends to the perception that weight discrimination is more socially acceptable and justifiable (often under the guise of “motivating” an individual to lose weight) than other forms, such as racial discrimination (Major et al. 2018; Papadopoulos and Brennan 2015; Pearl 2018). Therefore, individuals who face weight discrimination are especially likely to assign self-blame and exhibit shame for these experiences (Major and Schmader 2018; Schmitt et al. 2014).

In accordance with Abrutyn and Mueller’s (2014) theoretical proposition, we argue that weight discrimination may be a particularly robust contributor to suicidality, especially among adults in the age range we study, who have such a high likelihood of experiencing weight discrimination. Although the aforementioned study that investigated the relationship between discrimination and suicide ideation lends support for this argument (Hunger et al. 2020), it is limited in ways we seek to overcome. That study used small, select samples from crowdsourcing (n = 254) and behavioral research (n = 306) websites, which led the authors to note that results should be considered preliminary until replicated in a well-powered study. They also point to the need for studies utilizing larger samples to determine whether characteristics such as gender moderate the relationship between weight discrimination and suicidality. We make both of these research contributions in analysis of data from a large, more diverse nationally representative sample of U.S. adults. Our study is also the first to investigate the association between weight discrimination and suicide attempts among U.S. adults and whether gender moderates it.

We specifically investigate gender as a moderator because women face more stringent and unattainable body type expectations than men (Buote et al. 2011; Ciciurkaite and Perry 2018; Pingitore, Spring, and Garfield 1997). Women also face harsher societal judgment of their bodies (Ciciurkaite and Perry 2018; Frederick et al. 2022) and greater intolerance of their obesity (Pingitore et al. 1997).

Because of this, women are more likely than men to experience weight stigma and discrimination (Boswell and White 2015; Pearl et al. 2018; Puhl et al. 2008; Tomiyama et al. 2018) that is especially pronounced during the mid-30s to early 40s (Puhl et al. 2008). Women are also more likely to internalize weight bias (Boswell and White 2015; Himmelstein et al. 2017; Puhl et al. 2018), the belief that negative perceptions about body weight pertain to the self (Marshall, Latner, and Masuda 2020), and this has been linked to acute feelings of shame (Mensinger, Tylka, and Calamari 2018; Pearl and Puhl 2018). Given these factors, it is plausible that the relationship between weight discrimination and suicidality could be stronger among women than men.

## Data And Methods

### Data

We analyzed data from Add Health, a longitudinal nationally representative study of 7th to 12th graders in 1994 to 1995. During Wave I of data collection, 90,118 students were selected from 145 middle, junior, and high schools for participation in the in-school survey, and then 20,745 of those students were selected to complete an in-home survey (Harris 2013). A parent or guardian of these selected adolescents also completed a survey. Wave II data were collected from 14,738 Wave I respondents in 1996, with the exception of Wave I 12th graders and the Wave I disabled sample. Three subsequent waves of data were collected in 2001 to 2002 (Wave III), 2008 (Wave IV), and 2016 to 2018 (Wave V; including data from Wave I 12th graders). Wave III included 15,197 participants between the ages of 18 and 26, Wave IV included 15,701 participants between the ages of 24 and 32, and Wave V included 12,300 participants between the ages of 33 and 43. The Add Health surveys include a comprehensive set of questions regarding health status, health behaviors, peer networks, family composition and dynamics, romantic relationships, sexual activity, and more.

We used data from Wave V when participants were between the ages of 33 to 43 and data from Wave I (including from the parent/guardian survey). Wave V survey weights were used to account for Add Health’s complex survey design. Because we employed survey weights, we excluded 243 respondents missing data necessary to appropriately weight all study analyses in order to produce representative estimates.

We did not exclude any cases with missing data on our analytic variables because we used the Stata 17.0 *mi* function to handle missingness with multiple imputation, meaning our final analytic sample included 12,057 respondents. Imputed values replaced missing values that were randomly drawn from a posterior predictive distribution conditioned on the observed values (von Hippel 2020). The multiple imputation process averages the values of the parameter estimates across M samples to produce a single-point estimate and produces the standard errors through averaging the squared standard errors of the M estimates and calculating the variance of the M parameter estimates (Allison 2000). For our analyses, imputations were performed using “chained” equations over 10 iterations. Results from our imputed analyses were substantively similar to analyses that used listwise deletion.

### Measures

Our outcomes of interest were dichotomous indicators of past-year suicide ideation (1 = yes) and suicide attempts (1 = yes) based on the questions, “During the past 12 months, have you seriously thought about committing suicide?” and “During the past 12 months, how many times have you actually attempted suicide?” The responses to the latter question included “none,” “once,” “twice,” “three or four times,” and “five or more times.” Given the low number of suicide attempts, we created a dichotomous indicator by combining all the responses other than none into a single category (1 = yes).

Our primary independent variable was perceived interpersonal *weight discrimination* (1 = yes). Wave V of Add Health asked a series of questions from the Everyday Discrimination Scale (an abridged five-item version) that was developed by Williams et al. (1997). The Everyday Discrimination Scale is one of the most widespread instruments used to examine the relationship between perceived interpersonal discrimination and physical and mental health (Harnois et al. 2019). Questions on the abridged five-item version of the Everyday Discrimination Scale included “In your day-to-day life, how often have any of the following things happened to you?”: (1) you are treated with less courtesy or respect than other people, (2) you receive poorer service than other people at restaurants or stores, (3) people act as if they think you are not smart, (4) people act as if they are afraid of you, and (5) you are threatened or harassed. Responses included 1 = “never,” 2 = “rarely,” 3 = “sometimes,” and 4 = “often.” If respondents answered positively to at least one of the five questions, they were asked a follow-up question, “What do you think were the reasons why these experiences happened to you?” Answers included ancestry or national origin, biological sex, gender identity or gender expression, race, age, religion, weight, physical disability, an aspect of your physical appearance, sexual orientation, financial status, and “other.” Respondents could select as many or as few attributes as necessary. Any respondent that answered “yes” to weight as a reason was coded as 1, and any respondent that answered “no” was coded as 0. This dichotomous measure of weight discrimination has been used in myriad studies documenting a relationship between weight-based discrimination and physical and mental health (i.e., Andreyeva, Puhl, and Brownell 2008; Robinson et al. 2017; Schafer and Ferraro 2011; Sutin et al. 2016; Sutin, Stephan, and Terracciano 2015; Sutin and Terracciano 2013).

We controlled for *body mass index (BMI) category* by creating dummy variables based on BMI scores that were constructed from the respondents’ self-reported weight and height. We relied on self-reported weight and height because Add Health did not collect measured weight and height for the overall sample at Wave V. BMI weight categories reflected the Centers for Disease Control and Prevention’s guidelines for classifying individuals as “underweight” (BMI < 18.5), “healthy weight” (18.5 ≥ BMI ≤ 24.9), “overweight” (25.0 ≥ BMI ≤ 29.9), “obese class I” (30.0 ≥ BMI ≤ 34.9), “obese class II” (35.0 ≥ BMI ≤ 39.9), and “obese class III” (BMI ≥ 40.0). Given the low number of respondents (n = 107) that fell into the underweight category, we did not have the statistical power to differentiate between underweight and healthy weight and combined these categories into a single “not overweight” (reference) category (BMI ≤ 24.9). Although we recognize the limitations of BMI based on self-reports (Stommel and Schoenborn 2009) and the ability of BMI categories to capture weight statuses (Romero-Corral et al. 2008), they are both regularly used in national surveys to capture weight status.

We controlled for several mental and physical health indicators. We specifically accounted for mental health indicators that are associated with weight discrimination to determine if weight discrimination is associated with suicidality independent of them (Sutin et al. 2018).

We accounted for *depression* at Wave V (1 = yes) because it is among the strongest predictors of suicidality (Klonsky et al. 2016). This measure was constructed from responses to a modified five-item Center for Epidemiological Studies (CES-D) instrument that asked respondents the following: about how often in the past week they felt that they could not shake off the blues, felt depressed, were happy, felt sad, or felt that life was not worth living. We coded response options as 0 = “never or rarely,” 1 = “sometimes,” 2 = “a lot of the time,” and 3 = “most of the time or all of the time.” We reverse-coded Question 3 (were happy) and summed the five questions to derive depressive symptoms scores with a range of 0 to 15 (Cronbach’s α = .83). Higher scores indicated more depressive symptoms. A cutoff point of 20 coincides with diagnoses of depression when the original CES-D instrument is administered to individuals (Vilagut et al. 2016), and the cut point can be adjusted to 4 for the abbreviated five-item version (Hall et al. 2014; Lewinsohn et al. 1997).

We also controlled for a continuous indictor of *perceived stress* at Wave V (range 0–16) following Hatzenbuehler et al. (2009). The authors suggest that because weight discrimination is a well-established psychosocial stressor, we need to understand if the association between weight discrimination and mental health outcomes remains net of perceived stress. It was based on four Add Health items from the Perceived Stress Scale, a psychological instrument that measures perception of stress (Cohen, Kamarck, and Mermelstein 1983). Questions on the scale included: “In the past 30 days, how often have you felt that you were unable to control the important things in your life?”; “In the past 30 days, how often have you felt confident in your ability to handle your personal problems?”; “In the past 30 days, how often have you felt that things were going your way?”; and “In the past 30 days, how often have you felt that difficulties were piling up so high that you could not overcome them?” Responses included 0 = “never,” 1 = “almost never,” 2 = “sometimes,” 3 = “fairly often,” and 4 = “very often.” Questions 2 and 3 were reverse-coded, and responses to the four questions were summed (Cronbach’s α = .78).

Additionally, we controlled for *adolescent suicide ideation* (1 = yes) and *adolescent suicide attempts* (1 = yes) using Wave I measures. We included these controls because individuals who experienced suicidality in adolescence are at heightened risk of experiencing it in adulthood (Kessler et al. 2012).

*Fair/poor health* (1 = yes) at Wave V was also used as a control variable. It was created from the question, “In general, how is your health?” Responses included 1 = “excellent,” 2 = “very good,” 3 = “good,” 4 = “fair,” and 5 = “poor.” We combined “fair” and “poor” and “excellent,” “very good,” and “good” to create a dichotomous measure, which is typical (Graham and Fenelon 2023).

Additional controls included Wave V *educational attainment* (“less than high school” [reference], “high school degree or equivalent,” “some college,” and “bachelor’s degree or more”), *household income* (continuous measure), *marital status* (1 = married), and frequency of *social interactions* (range = 1–7). Wave I adolescent family socioeconomic status (*mother’s educational attainment*: “less than high school” [reference], “high school degree or equivalent,” “some college,” and “bachelor’s degree or more”) and *adolescent household income* (continuous measure) were also controlled. Finally, we controlled for demographic indicators of *race-ethnicity* (coded according to the Add Health guidelines as “non-Hispanic, White” [reference]; “non-Hispanic, Black”; “Hispanic”; “non-Hispanic, Asian/Pacific Islander”; and “non-Hispanic, Other”), Wave V *age* (range = 33–43), *nativity* (1 = foreign-born), and *gender* (1 = female).

### Analytic Strategy

We used Stata Version 17.0 (StataCorp, College Station, Texas) for our analyses. We first estimated the weighted descriptive statistics for our overall sample and stratified by gender to contextualize our findings. Next, we estimated the associations between weight discrimination and each of our outcomes through a series of weighted logistic regression models. We first estimated the bivariate relationship between weight discrimination and our outcomes unadjusted for our control variables to determine if initial associations existed (Model 1). Next, we estimated a model with both weight discrimination and BMI category (Model 2) to determine if weight discrimination was associated with suicidality net of weight classification. Then we added all of our other control variables to the model (Model 3). Finally, we added an interaction term between gender and weight discrimination to this fully adjusted model (Model 4).

In line with current methodological recommendations for examining interactions in nonlinear models that suggest that the coefficient from the interaction term in nonlinear models should not be used to draw conclusions about the significance of the interaction (Buis 2010; Hullenaar and Frisco 2020; Mize 2019; Mustillo, Lizardo, and McVeigh 2018), we followed Mize’s (2019) guidelines for estimating and interpreting nonlinear interaction effects to determine if gender is a moderator of the relationship between weight discrimination and each outcome. Accordingly, using Model 4, we first estimated and plotted the predicted probabilities of each outcome for the four combinations of the weight discrimination and gender variables. Then, to test for an interactive effect of weight discrimination and gender, we estimated the first differences (marginal effects or Δ) and second differences (test of interaction) in these probabilities along with the 95% confidence intervals (CIs).

## Results

### Descriptive Statistics

Table 1 presents descriptive statistics. For brevity, we limit discussion of them to our indicators of suicidality, weight discrimination, and weight categories. Overall, suicide ideation was relatively rare in this sample. Less than 7% of respondents indicated they had past-year suicide ideation. A slightly larger proportion of men (7.1%) than women (6.5%) experienced suicide ideation, but this difference is not significant. Suicide attempts were even rarer, given that only 1.7% of respondents had attempted suicide in the past year in the overall sample, and the difference between men and women is negligible.

**Table 1.** Weighted Descriptive Statistics for Full Sample and by Gender (*N* = 12,057).

	Full Sample		Men		Women		Gender Difference
	*N* = 12,057		*n* = 5,239		*n* = 6,818		
	% or Mean	SE	% or Mean	SE	% or Mean	SE	Significance
Wave V							
Suicide ideation (1 = yes)	6.80		7.09		6.51		
Suicide attempts (1 = yes)	1.68		1.67		1.70		
Weight discrimination (1 = yes)	12.63		10.18		15.10		***
BMI category							
Not overweight (reference)	27.14		21.78		32.58		***
Overweight	31.98		38.49		25.38		***
Obese class I	21.46		22.85		20.05		**
Obese class II	10.88		10.30		11.46		
Obese class III	8.54		6.59		10.52		***
Depression (1 = yes)	24.88		23.26		26.52		**
Perceived stress (range = 0–16)	5.05	.05	4.83	.06	5.28	.06	***
Fair/poor health (1 = yes)	14.13		14.99		13.25		
Educational attainment							
Less than high school (reference)	5.55		6.71		4.38		***
High school degree or equivalent	16.51		20.09		12.88		***
Some college	41.35		41.15		41.54		
Bachelor’s degree or more	36.59		32.05		41.19		***
Household income (range = 1–13)	8.81	.10	9.01	.11	8.61	.11	***
Married (1 = yes)	57.74		56.47		59.03		
Social interactions (range = 1–7)	5.06	.03	5.08	.03	5.05	.03	
Wave I							
Adolescent suicide ideation (1 = yes)	13.61		10.95		16.30		***
Adolescent suicide attempts (1 = yes)	3.87		2.17		5.60		***
Mother’s educational attainment							
Less than high school (reference)	15.40		14.32		16.49		
High school degree or equivalent	28.45		28.45		28.45		
Some college	33.84		34.52		33.14		
Bachelor’s degree or more	22.32		22.72		21.92		
Adolescent household income (in thousands of dollars)	46.18	1.69	46.40	1.84	45.95	1.82	
Demographics							
Race-ethnicity							
Non-Hispanic, White (reference)	66.53		66.72		66.33		
Non-Hispanic, Black	15.12		14.49		15.76		
Hispanic	11.34		11.38		11.31		
Non-Hispanic, Asian/Pacific Islander	3.69		3.87		3.52		
Non-Hispanic, other	3.32		3.55		3.08		
Female (1 = yes)	49.67						
Age (range = 33–43 years)	37.87	.12	37.98	.12	37.76	.12	***
Foreign born (1 = yes)	5.99		5.92		6.05		

*Source:* National Longitudinal Study of Adolescent to Adult Health.

*Note:* BMI = body mass index; SE = standard error. The *p* values are from *t* test or Pearson’s chi-square test.

** *p* < .01, ***p < .001.

Approximately 13% of the total sample experienced weight discrimination, but a significantly larger proportion of women reported weight discrimination (15.1%) than men (10.2%), corroborating prior research findings (e.g., Puhl et al. 2008). Supplementary analyses indicated that 5.3% of individuals who were in the not overweight (BMI ≤ 24.9) reference category reported weight discrimination compared to 5.2%, 14.3%, 26.5%, and 42.7%, respectively, of individuals who were in the overweight (25.0 ≥ BMI ≤ 29.9), obese class I (30.0 ≥ BMI ≤ 34.9), obese class II (35.0 ≥ BMI ≤ 39.9), and obese class III (BMI ≥ 40.0) categories

In terms of proportions of respondents in each BMI category, less than a third of respondents were in the not overweight category (27.1%), approximately 32% were in the overweight category, and the remainder were in the obese class I (21.5%), obese class II (10.9%), or obese class III (8.5%) categories. However, the proportions of respondents in each category differed between men and women. A significantly larger proportion of women (32.6%) were in the not overweight category than men (21.8%), whereas a significantly larger proportion of men (38.5%) were in the overweight category than women (25.4%). Moreover, although a significantly larger proportion of men (22.9%) than women (20.1%) fell into the obese class I category, a significantly larger proportion of women (10.5%) were in the obese class III category than men (6.6%).

### Weight Discrimination, Suicide Ideation, and Suicide Attempts

Table 2 presents results from models estimating the relationship between weight discrimination and suicide ideation. The bivariate model (Model 1) indicates that a robust and significant positive association exists between weight discrimination and suicide ideation without accounting for control variables (odds ratio [OR] = 2.42; 95% CI: 1.93, 3.05). Model 2 controls for BMI category. Weight discrimination has a large, positive, and significant association with suicide ideation (OR = 2.72; 95% CI: 2.09, 3.53), whereas overweight and obesity do not. Model 3 adjusts for all of our other control variables. Weight discrimination is still significantly associated with suicide ideation. Individuals who reported weight discrimination have odds of suicide ideation that are higher than their counterparts who did not report weight discrimination by a factor of 1.82.

**Table 2.** Weighted Estimates (Odds Ratios) from Logistic Regression Models Predicting Suicide Ideation (*N* = 12,057).

	Model 1		Model 2		Model 3		Model 4	
	OR	95% CI	OR	95% CI	OR	95% CI	OR	95% CI
Weight discrimination (1 = yes)	2.42***	(1.93, 3.05)	2.72***	(2.09, 3.53)	1.82***	(1.35, 2.46)	2.17**	(1.37, 3.42)
BMI category (reference = not overweight)								
Overweight			.89	(.69, 1.14)	.98	(.72, 1.33)	1.00	(.73, 1.35)
Obese class I			.92	(.68, 1.23)	.89	(.63, 1.25)	.90	(.64, 1.27)
Obese class II			.66*	(.46, .95)	.60*	(.38, 0.97)	.61*	(.38, .98)
Obese class III			.69	(.44, 1.07)	.63	(.38, 1.04)	.63	(.38, 1.05)
Depression (1 = yes)					6.65***	(5.04, 8.78)	6.64***	(5.04, 8.75)
Perceived stress					1.23***	(1.18, 1.29)	1.23***	(1.18, 1.29)
Adolescent suicide ideation (1 = yes)					1.96***	(1.40, 2.74)	1.98***	(1.41, 2.77)
Adolescent suicide attempts (1 = yes)					1.04	(.57, 1.89)	1.03	(.57, 1.88)
Fair/poor health (1 = yes)					1.30	(1.00, 1.69)	1.29	(.99, 1.69)
Educational attainment (reference = < high school)								
High school degree or equivalent					.55*	(.34, .88)	.55*	(.34, .88)
Some college					.92	(.58, 1.47)	.93	(.58, 1.47)
Bachelor’s degree or more					.92	(.56, 1.51)	.92	(.56, 1.51)
Household income					1.05*	(1.00, 1.10)	1.05*	(1.00, 1.10)
Married (1 = yes)					.60***	(.48, .74)	.60***	(.49, .74)
Social interactions					.91*	(.84, .98)	.91*	(.84, .98)
Mother’s educational attainment (reference = < high school)								
High school degree or equivalent					1.02	(.68, 1.54)	1.02	(.68, 1.53)
Some college					.89	(.58, 1.36)	.88	(.58, 1.35)
Bachelor’s degree or more					1.12	(.70, 1.79)	1.12	(.70, 1.79)
Adolescent household income					.99*	(.99, 1.00)	.99*	(.99, 1.00)
Race-ethnicity (reference = non-Hispanic, White)								
Non-Hispanic, Black					.82	(.58, 1.14)	.81	(.58, 1.14)
Hispanic					.88	(.56, 1.38)	.88	(.56, 1.37)
Non-Hispanic, Asian/Pacific Islander					1.00	(.55, 1.82)	1.01	(.55, 1.83)
Non-Hispanic, other					2.22**	(1.34, 3.70)	2.22**	(1.33, 3.69)
Age					.91**	(.86, .97)	.91**	(.86, .97)
Foreign born (1 = yes)					.99	(.58, 1.66)	.98	(.58, 1.66)
Female (1 = yes)					.68**	(.53, .87)	.73*	(.54, .97)
Female × Weight discrimination							.73	(.40, 1.34)

*Source:* National Longitudinal Study of Adolescent to Adult Health.

*Note:* BMI = body mass index; OR = odds ratio; CI = confidence interval.

* *p* < .05, **p < .01, ***p < .001.

Results of the predicted probabilities of suicide ideation for the four combinations of the weight discrimination and gender categories estimated from Model 4 reveal that both men and women who experienced weight discrimination have significantly higher probabilities than their male and female counterparts who did not experience weight discrimination (respectively); however, the second difference is not significant, indicating that the effect of weight discrimination on suicide ideation does not significantly differ between men and women (see Appendix Figure A1 in the online version of the article). In other words, we did not find that gender moderated the relationship between weight discrimination and suicide ideation.

Table 3 presents results from models estimating the relationship between weight discrimination and suicide attempts. Similar to suicide ideation, weight discrimination has a robust and significant positive association with suicide attempts in the bivariate model (Model 1; OR = 3.50; 95% CI: 2.26, 5.40), and this association remains after adjusting for BMI category in Model 2 (OR = 3.28; 95% CI: 1.87, 5.75). Overweight and obesity are also not significantly associated with suicide attempts. After adjusting for all control variables (Model 3), weight discrimination is associated with significantly higher estimated odds of suicide attempts relative to no weight discrimination (OR = 2.24; 95% CI: 1.26, 3.99). Similar to suicide ideation, estimates of the predicted probabilities of suicide attempts by gender and weight discrimination from Model 4 (see Appendix Figure A2 in the online version of the article) show that there is not a significant interaction effect between gender and weight discrimination.

**Table 3.** Weighted Estimates (Odds Ratios) from Logistic Regression Models Predicting Suicide Attempts (*N* = 12,057).

	Model 1		Model 2		Model 3		Model 4	
	OR	95% CI	OR	95% CI	OR	95% CI	OR	95% CI
Weight discrimination (1 = yes)	3.50***	(2.26, 5.40)	3.28***	(1.87, 5.75)	2.24**	(1.26, 3.99)	2.76*	(1.11, 6.85)
BMI category (reference = not overweight)								
Overweight			.68	(.35, 1.34)	.68	(.34, 1.36)	.69	(.35, 1.37)
Obese class I			.99	(.53, 1.85)	.94	(.49, 1.80)	.95	(.50, 1.81)
Obese class II			1.07	(.49, 2.32)	.93	(.39, 2.23)	.94	(.40, 2.22)
Obese class III			.92	(.32, 2.58)	.83	(.27, 2.57)	.83	(.27, 2.55)
Depression (1 = yes)					3.15***	(1.87, 5.31)	3.14***	(1.88, 5.26)
Perceived stress					1.21***	(1.12, 1.31)	1.21***	(1.12, 1.31)
Adolescent suicide ideation (1 = yes)					1.63	(.96, 2.78)	1.65	(.98, 2.79)
Adolescent suicide attempts (1 = yes)					1.44	(.67, 3.11)	1.45	(.67, 3.11)
Fair/poor health (1 = yes)					.89	(.54, 1.49)	.89	(.53, 1.48)
Educational attainment (reference = less thanhigh school)								
High school degree or equivalent					.73	(.40, 1.36)	.74	(.40, 1.36)
Some college					.54	(.29, 1.01)	.54	(.29, 1.01)
Bachelor’s degree or more					.31*	(.12, .79)	.31*	(.12, .79)
Household income					.99	(.91, 1.08)	.99	(.91, 1.08)
Married (1 = yes)					.60*	(.37, .97)	.59*	(.36, .97)
Social interactions					.84*	(.73, .97)	.84*	(.73, .97)
Mother’s educational attainment (reference = lessthan high school)								
High school degree or equivalent					.71	(.34, 1.50)	.70	(.33, 1.48)
Some college					.79	(.39, 1.62)	.79	(.40, 1.61)
Bachelor’s degree or more					.61	(.24, 1.59)	.62	(.24, 1.56)
Adolescent household income					1.00	(1.00, 1.01)	1.00	(1.00, 1.01)
Race-ethnicity (reference = non-Hispanic, White)								
Non-Hispanic, Black					2.14**	(1.26, 3.66)	2.14**	(1.25, 3.65)
Hispanic					1.31	(.65, 2.64)	1.30	(0.64, 2.64)
Non-Hispanic, Asian/Pacific Islander					3.79**	(1.46, 9.85)	3.81**	(1.47, 9.86)
Non-Hispanic, other					2.11	(.97, 4.57)	2.12	(.98, 4.59)
Age					.88	(.76, 1.00)	.88	(.76, 1.00)
Foreign born (1 = yes)					1.85	(.76, 4.50)	1.86	(.76, 4.55)
Female (1 = yes)					.76	(.46, 1.25)	.85	(.48, 1.49)
Female × Weight discrimination							.68	(.25, 1.89)

*Source:* National Longitudinal Study of Adolescent to Adult Health.

*Note:* BMI = body mass index; OR = odds ratio; CI = confidence interval.

* *p* < .05, **p < .01, ***p < .001.

### Supplementary Analyses

In supplementary analyses, we restricted analysis to the sample of respondents that fell into the overweight and obese categories because so few individuals (5.3%) in the not overweight category reported weight discrimination (n = 8,614). As such, these models focus more explicit attention on men and women likely to experience and perceive weight discrimination. As shown in Appendix Tables A1 and A2 in the online version of the article, estimates from logistic regression models predicting both suicide ideation and suicide attempts were comparable to estimates using the full sample, but gender differences in the association between weight discrimination and suicide ideation are pronounced in the subsample of individuals with overweight or obesity. We found that gender significantly moderated the relationship between weight discrimination and suicide ideation (Appendix Figure A3 in the online version of the article). Contrary to expectation, the effect of weight discrimination on suicide ideation is significantly greater among men than women.

## Discussion

The purpose of our study was to investigate the relationships between perceived interpersonal weight discrimination and two dimensions of suicidality—suicide ideation and suicide attempts—among a nationally representative sample of U.S. adults ages 33 to 43. We also examined whether gender moderated these relationships. To our knowledge, this is the first large-scale nationally representative study of weight discrimination and multiple dimensions of suicidality among U.S. adults and the first to consider gender differences in these associations. Our focus on men and women in the age range that we study is especially pertinent given the degree to which they experience suicidality (National Institute of Mental Health 2022) and weight discrimination (Puhl et al. 2008) relative to adults in other age ranges.

Our study’s first contribution is building on the work of Hunger et al. (2020) by demonstrating that weight discrimination is positively associated with suicide ideation in a large national sample of U.S. adults net of BMI categories, depression, perceived stress, and suicidality earlier in the life course. As such, our study demonstrates the representativeness and the robustness of the weight discrimination–suicide ideation relationship.

Our second study contribution, and one of significant import, is showing that weight discrimination is not only related to suicidal thoughts. We are the first to show that weight discrimination is also associated with increased odds of attempting suicide among U.S. adults. The importance of identifying a relationship between weight discrimination and both suicide ideation and attempts cannot be understated. These two dimensions of suicidality are different phenomena (Klonsky et al. 2016), and suicide attempts are a significantly greater risk factor than suicide ideation for future suicide ideation, attempts, and mortality (Parra-Uribe et al. 2017).

We also investigated whether there were gender differences in these relationships. We expected to find that the association between weight discrimination and suicidality would be stronger among women than men because they face starker societal criticism of their bodies (Ciciurkaite and Perry 2018; Frederick et al. 2022), more weight stigma and discrimination (Boswell and White 2015; Pearl et al. 2018; Tomiyama et al. 2018), and greater internalization of weight bias (Boswell and White 2015; Himmelstein et al. 2017; Puhl et al. 2018). However, contrary to expectation, we found that the association between weight discrimination and the dimensions of suicidality that we investigated were similar for men and women in the overall study sample despite the fact that women faced more weight discrimination. This does not appear to translate into women who perceive weight discrimination having a higher risk of suicide ideation and attempts than men.

These findings suggest that weight discrimination is pernicious, likely due to societal perceptions of the controllability of body weight (Major et al. 2012; Schmitt et al. 2014) and broad societal forces that blame individuals for their body weight and size (Papadopoulos and Brennan 2015; Ringel and Ditto 2019; Saguy 2013). This produces greater societal acceptability and embracement of weight discrimination relative to discrimination due to other attributes (i.e., racial discrimination; Major et al. 2018; Papadopoulos and Brennan 2015; Pearl 2018), which may result in men and women being equally likely to evaluate weight discrimination as legitimate, deserved, worthy of self-blame, and shame (Major and Schmader 2018; Schmitt et al. 2014).

However, it is important to note that in our supplementary analyses, we found a significant moderating effect of gender on suicide ideation when our sample was restricted to men and women classified as overweight or obese. The effect of weight discrimination was stronger among men than women. Although we cannot be certain as to why this is the case, we speculate that it is possible that there are other psychosocial factors at play that may contribute to this finding. Women are more likely than men to employ active coping responses (i.e., talking to others about their experiences) in the face of interpersonal discriminatory experiences (Polanco-Roman, Danies, and Anglin 2016), whereas men become less extraverted than women in response to discrimination (Kim, Song, and Sutin 2021). These gendered responses likely reflect the pervasive feminization of social support in the United States (Reevy and Maslach 2001), which may undermine men’s willingness to seek social support in the face of weight discrimination. It is possible that men who are classified as having overweight and obesity are less likely to seek social support than their female peers, contributing to the greater effect of weight discrimination on suicidal thoughts among men than women. Exploring this possibility, among others, is outside the scope of our study, but we encourage future research to do so.

Of final note regarding the implications of these findings, the broader literature on interpersonal discrimination and suicidality has provided evidence that interpersonal discrimination based on other attributes such as race-ethnicity (Coimbra et al. 2022) and LGBTQ or sexual minority status (Layland et al. 2020; Sutter and Perrin 2016) are associated with suicidality in select samples of U.S. adults. We suggest that future research could explore whether weight discrimination has a stronger association with suicidality relative to these other forms in order to investigate the proposition that the perceived controllability of a stigma matters to the likelihood of suicidal thoughts and behaviors. Based on theoretical framework, we anticipate that it could.

Our study’s contributions must be considered within the context of some limitations. First, weight discrimination and suicidality are both measured at Wave V, meaning that we can only speak to the association between the two and not causality. It is possible there is a bidirectional association in which suicidal adults are more likely to perceive interpersonal weight discrimination. Second, because we examine this relationship within a specific age range, we cannot be sure whether this relationship extends to younger or older adults. Research should consider this relationship among other adult age groups. Third, our weight discrimination measure does not assess the frequency, intensity, or timing of discrimination due to limitations with how the Add Health study asks about frequency of discriminatory experiences relative to different forms of discrimination. It is possible the extent to which one experiences weight discrimination matters to the likelihood of suicidality. Finally, weight discrimination is a subjective measure. Perceptions of what constitutes weight discrimination among study respondents may vastly differ and bias study findings.

Limitations notwithstanding, our findings contribute to the body of literature that underscores the impact of weight stigma and discrimination on suicidality. In a nationally representative sample of U.S. adults in an age range with a higher prevalence of both weight discrimination and suicidality, we find that weight discrimination is positively associated with both suicide ideation and suicide attempts, and these associations were equally salient among men and women within our overall sample. Moreover, our findings indicated that weight discrimination but not body weight itself was related to suicidality, which lends further support for scholars’ speculations and findings that other factors associated with body weight are more relevant to suicidality than body weight itself (i.e., Eisenberg et al. 2003; Haynes et al. 2019; Sutin et al. 2018). Overall, these findings strongly emphasize the need for policies and initiatives to address issues of weight stigma and discrimination within our society. As Tomiyama et al. (2018) suggest, societal-level approaches could include refocusing public health messaging from blame and shame to promoting healthy behaviors without mentioning weight or size and implementing legal protection against weight-based discrimination. Such broader level approaches would have far-reaching impacts on helping to destigmatize body weight and eradicate weight-based discrimination, including on an interpersonal level, ultimately helping to alleviate an important risk factor for suicidality.

## Supplemental Material

sj-docx-1-hsb-10.1177_00221465231200634 – Supplemental material for The Mental “Weight” of Discrimination: The Relationship between Perceived Interpersonal Weight Discrimination and Suicidality in the United States Click here for additional data file. Supplemental material, sj-docx-1-hsb-10.1177_00221465231200634 for The Mental “Weight” of Discrimination: The Relationship between Perceived Interpersonal Weight Discrimination and Suicidality in the United States by Carlyn E. Graham and Michelle L. Frisco in Journal of Health and Social Behavior
