# Supplementary material for: The Mental “Weight” of Discrimination: The Relationship between Perceived Interpersonal Weight Discrimination and Suicidality in the United States
Source: J Health Soc Behav. 2023 Sep 30;64(4):610–25. doi: 10.1177/00221465231200634 (PMC10683344; doi:10.1177/00221465231200634)
Supplement: sj-docx-1-hsb-10.1177_00221465231200634 – Supplemental material for The Mental “Weight” of Discrimination: The Relationship between Perceived Interpersonal Weight Discrimination and Suicidality in the United States [file sj-docx-1-hsb-10.1177_00221465231200634.docx]

**Journal** of **Health**

and **Social Behavior**

OFFICIAL JOURNAL OF THE AMERICAN SOCIOLOGICAL ASSOCIATION

**ONLINE SUPPLEMENT**

**to article in**

Journal of Health and Social Behavior

**The Mental “Weight” of Discrimination: The Relationship between Perceived Interpersonal Weight Discrimination and Suicidality in the United States**

**Carlyn E. Graham**

*The Pennsylvania State University*

**Michelle L. Frisco**

*The Pennsylvania State University*

**Appendix**

Table A1. Weighted estimates (odds ratios) from logistic regression models predicting suicide ideation—overweight and obese subsample (N=8,614)

|  | Model 1 | | Model 2 | | Model 3 | | Model 4 | |
| --- | --- | --- | --- | --- | --- | --- | --- | --- |
|  | OR | 95% CI | OR | 95% CI | OR | 95% CI | OR | 95% CI |
| Weight discrimination (1=yes) | 2.52*** | (1.95, 3.25) | 2.79*** | (2.08, 3.73) | 1.69** | (1.21, 2.38) | 2.35** | (1.45, 3.82) |
| BMI category (ref=not overweight) |  |  |  |  |  |  |  |  |
| Obese class I |  |  | 1.02 | (0.76, 1.37) | 0.96 | (0.71, 1.30) | 0.95 | (0.71, 1.28) |
| Obese class II |  |  | 0.74 | (0.52, 1.05) | 0.64 | (0.41, 1.02) | 0.63* | (0.40, 1.00) |
| Obese class III |  |  | 0.77 | (0.50, 1.18) | 0.69 | (0.42, 1.12) | 0.67 | (0.42, 1.10) |
| Depression (1=yes) |  |  |  |  | 5.89*** | (4.05, 8.57) | 5.85*** | (4.04, 8.48) |
| Perceived stress |  |  |  |  | 1.25*** | (1.18, 1.32) | 1.26*** | (1.18, 1.33) |
| Adolescent suicide ideation (1=yes) |  |  |  |  | 1.74* | (1.15, 2.65) | 1.76** | (1.16, 2.69) |
| Adolescent suicide attempts (1=yes) |  |  |  |  | 1.08 | (0.48, 2.44) | 1.08 | (0.48, 2.41) |
| Fair/poor health (1=yes) |  |  |  |  | 1.40* | (1.04, 1.89) | 1.40* | (1.03, 1.89) |
| Educational attainment (ref=< high school) |  |  |  |  |  |  |  |  |
| High school degree or equivalent |  |  |  |  | 0.48* | (0.27, 0.86) | 0.49* | (0.27, 0.87) |
| Some college |  |  |  |  | 0.72 | (0.43, 1.20) | 0.72 | (0.43, 1.20) |
| Bachelor's degree or more |  |  |  |  | 0.71 | (0.40, 1.27) | 0.72 | (0.40, 1.28) |
| Household income |  |  |  |  | 1.07* | (1.01, 1.13) | 1.07* | (1.01, 1.13) |
| Married (1=yes) |  |  |  |  | 0.63** | (0.48, 0.82) | 0.63** | (0.48, 0.82) |
| Social interactions |  |  |  |  | 0.95 | (0.87, 1.05) | 0.95 | (0.87, 1.05) |
| Mother's educational attainment (ref=< high school) | |  |  |  |  |  |  |  |
| High school degree or equivalent |  |  |  |  | 1.19 | (0.71, 1.99) | 1.17 | (0.70, 1.95) |
| Some college |  |  |  |  | 0.84 | (0.52, 1.37) | 0.83 | (0.52, 1.35) |
| Bachelor's degree or more |  |  |  |  | 1.21 | (0.73, 2.01) | 1.21 | (0.74, 1.99) |
| Adolescent household income |  |  |  |  | 1.00 | (0.99, 1.00) | 1.00 | (0.99, 1.00) |
| Race/ethnicity (ref=non-Hispanic, White) |  |  |  |  |  |  |  |  |
| Non-Hispanic, Black |  |  |  |  | 0.69 | (0.45, 1.06) | 0.68 | (0.44, 1.04) |
| Hispanic |  |  |  |  | 0.87 | (0.55, 1.37) | 0.86 | (0.55, 1.35) |
| Non-Hispanic, Asian/Pacific Islander |  |  |  |  | 1.05 | (0.51, 2.19) | 1.07 | (0.52, 2.21) |
| Non-Hispanic, Other |  |  |  |  | 1.46 | (0.82, 2.61) | 1.45 | (0.82, 2.57) |
| Age |  |  |  |  | 0.90* | (0.83, 0.97) | 0.90** | (0.83, 0.97) |
| Foreign-born (1=yes) |  |  |  |  | 1.26 | (0.67, 2.38) | 1.27 | (0.67, 2.41) |
| Female (1=yes) |  |  |  |  | 0.73* | (0.55, 0.98) | 0.86 | (0.62, 1.18) |
| Female x weight discrimination |  |  |  |  |  |  | 0.55 | (0.29 1.03) |
| ***p<0.001 **p<0.01 *p<0.05 |  |  |  |  |  |  |  |  |
| *Source: National Longitudinal Study of Adolescent to Adult Health* | | |  |  |  |  |  |  |

Table A2. Weighted estimates (odds ratios) from logistic regression models predicting suicide attempts—overweight and obese subsample (N=8,614)

|  | Model 1 | | Model 2 | | Model 3 | | Model 4 | |
| --- | --- | --- | --- | --- | --- | --- | --- | --- |
|  | OR | 95% CI | OR | 95% CI | OR | 95% CI | OR | 95% CI |
| Weight discrimination (1=yes) | 3.74*** | (2.25, 6.21) | 3.37*** | (1.82, 6.25) | 2.13* | (1.16, 3.93) | 2.50 | (0.90, 6.99) |
| BMI category (ref=not overweight) |  |  |  |  |  |  |  |  |
| Obese class I |  |  | 1.41 | (0.77, 2.58) | 1.41 | (0.82, 2.45) | 1.40 | (0.81, 2.41) |
| Obese class II |  |  | 1.61 | (0.80, 3.25) | 1.50 | (0.70, 3.23) | 1.48 | (0.69, 3.18) |
| Obese class III |  |  | 1.29 | (0.56, 2.96) | 1.29 | (0.54, 3.11) | 1.27 | (0.52, 3.13) |
| Depression (1=yes) |  |  |  |  | 4.25*** | (2.24, 8.04) | 4.23*** | (2.24, 7.99) |
| Perceived stress |  |  |  |  | 1.20*** | (1.09, 1.31) | 1.20*** | (1.10, 1.31) |
| Adolescent suicide ideation (1=yes) |  |  |  |  | 2.02* | (1.12, 3.66) | 2.03* | (1.13, 3.66) |
| Adolescent suicide attempts (1=yes) |  |  |  |  | 1.00 | (0.39, 2.59) | 1.01 | (0.39, 2.59) |
| Fair/poor health (1=yes) |  |  |  |  | 0.84 | (0.47, 1.48) | 0.84 | (0.47, 1.48) |
| Educational attainment (ref=less than high school) |  |  |  |  |  |  |  |  |
| High school degree or equivalent |  |  |  |  | 0.59 | (0.28, 1.26) | 0.60 | (0.28, 1.26) |
| Some college |  |  |  |  | 0.48* | (0.24, 0.96) | 0.48* | (0.24, 0.95) |
| Bachelor's degree or more |  |  |  |  | 0.19* | (0.06, 0.67) | 0.19* | (0.06, 0.67) |
| Household income |  |  |  |  | 1.04 | (0.95, 1.14) | 1.04 | (0.95, 1.14) |
| Married (1=yes) |  |  |  |  | 0.59 | (0.33, 1.03) | 0.58 | (0.33, 1.03) |
| Social interactions |  |  |  |  | 0.92 | (0.79, 1.09) | 0.92 | (0.79, 1.09) |
| Mother's educational attainment (ref=less than high school) | |  |  |  |  |  |  |  |
| High school degree or equivalent |  |  |  |  | 0.70 | (0.32, 1.53) | 0.69 | (0.32, 1.51) |
| Some college |  |  |  |  | 0.62 | (0.29, 1.35) | 0.62 | (0.29, 1.34) |
| Bachelor's degree or more |  |  |  |  | 0.75 | (0.27, 2.13) | 0.76 | (0.27, 2.14) |
| Adolescent household income |  |  |  |  | 1.00 | (0.99, 1.01) | 1.00 | (0.99, 1.01) |
| Race/ethnicity (ref=non-Hispanic, White) |  |  |  |  |  |  |  |  |
| Non-Hispanic, Black |  |  |  |  | 2.30* | (1.12, 4.70) | 2.28* | (1.11, 4.67) |
| Hispanic |  |  |  |  | 1.09 | (0.48, 2.50) | 1.09 | (0.48, 2.49) |
| Non-Hispanic, Asian/Pacific Islander |  |  |  |  | 4.65* | (1.41, 15.33) | 4.66* | (1.42, 15.27) |
| Non-Hispanic, Other |  |  |  |  | 2.51* | (1.04, 6.06) | 2.53* | (1.04, 6.12) |
| Age |  |  |  |  | 0.89 | (0.75, 1.05) | 0.89 | (0.75, 1.05) |
| Foreign-born (1=yes) |  |  |  |  | 2.66 | (0.96, 7.35) | 2.68 | (0.98, 7.37) |
| Female (1=yes) |  |  |  |  | 0.93 | (0.53, 1.62) | 1.02 | (0.50, 2.07) |
| Female x weight discrimination |  |  |  |  |  |  | 0.76 | (0.25, 2.30) |
| ***p<0.001 **p<0.01 *p<0.05 |  |  |  |  |  |  |  |  |
| *Source: National Longitudinal Study of Adolescent to Adult Health* | | |  |  |  |  |  |  |

Figure A1. Predicted probability of suicide ideation by weight discrimination and gender (N=12,057)


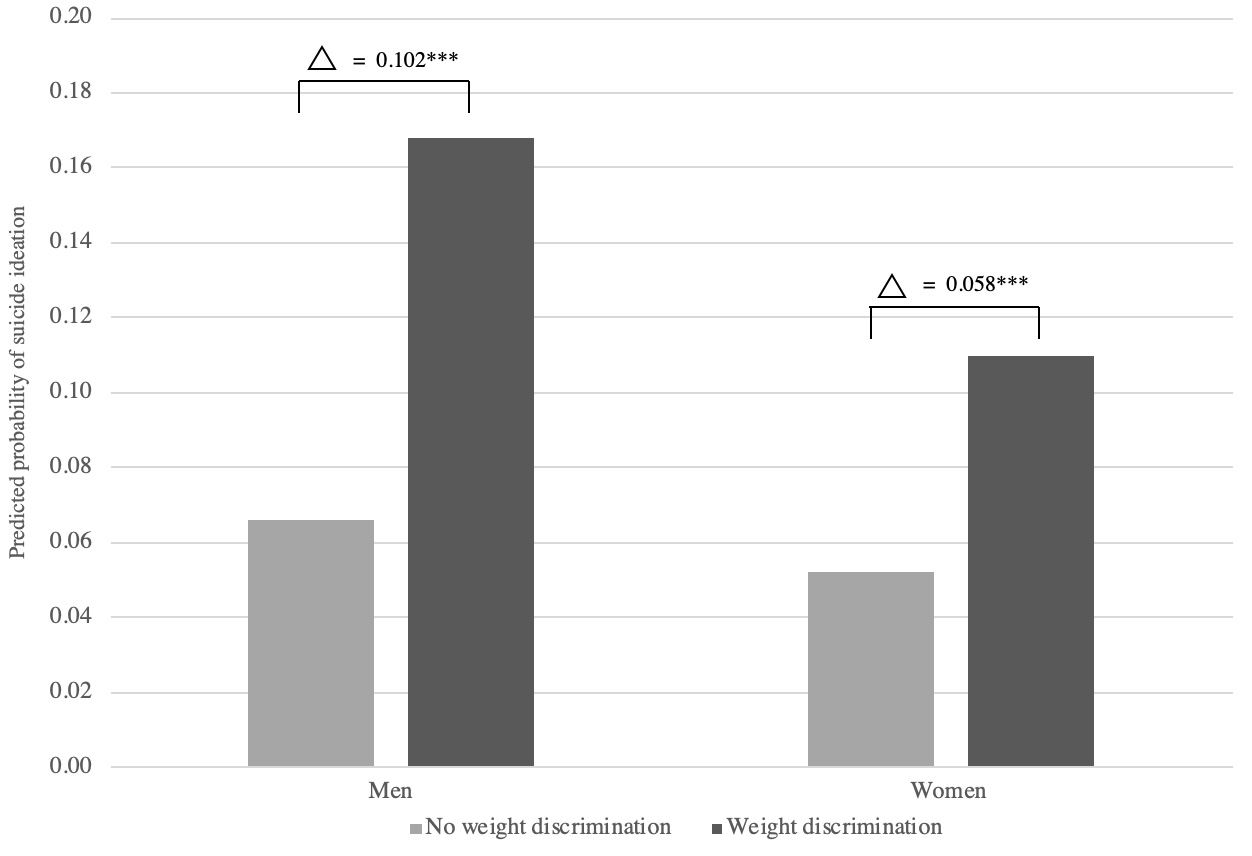


Δ = 0.058* between men, weight discrimination and women, weight discrimination

Δ = 0.014* between men, no weight discrimination and women, no weight discrimination

Second differences = 0.044, *p* = 0.098

*Source: National Longitudinal Study of Adolescent to Adult Health*

Figure A2. Predicted probability (standard errors) of suicide attempt by weight discrimination and gender (N=12,057)


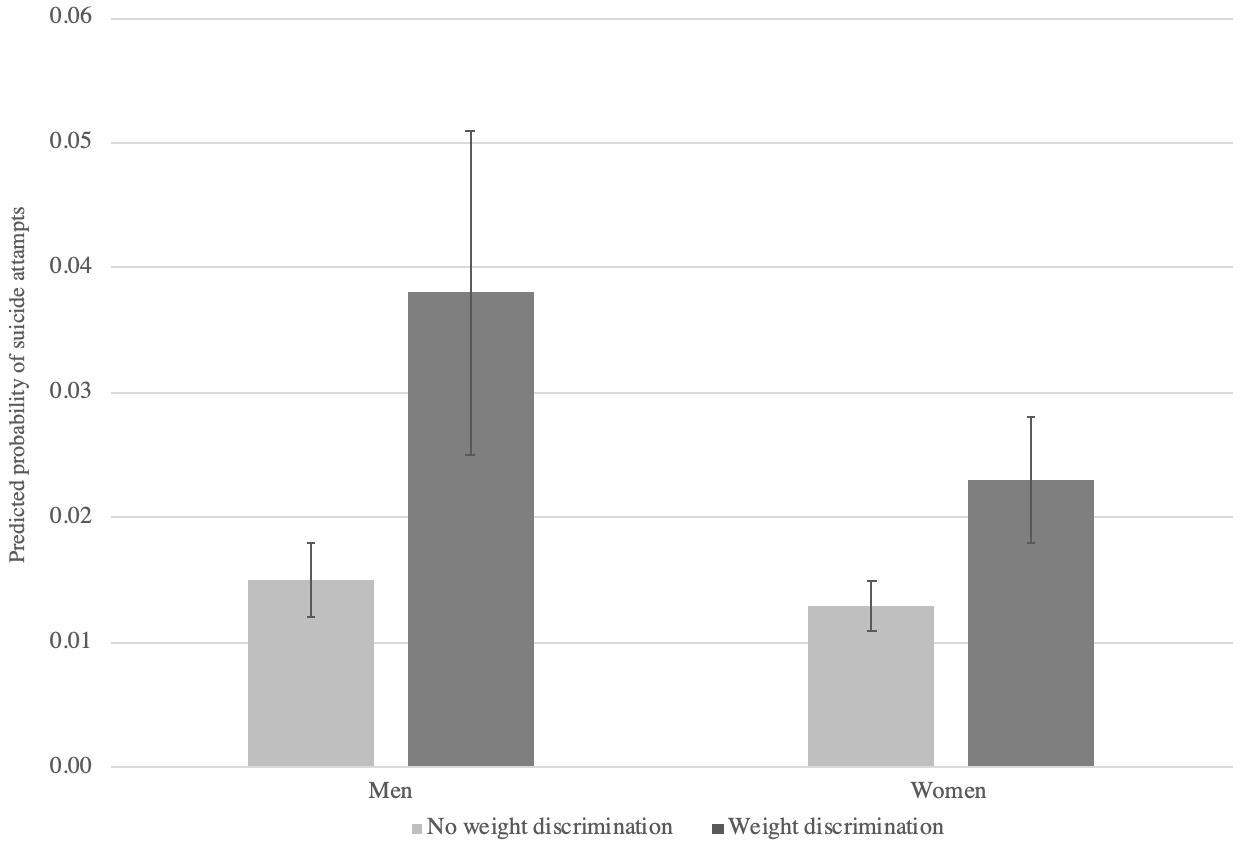


Note: there are no significant differences (Δ) at p<0.05 level between any of the categories

*Source: National Longitudinal Study of Adolescent to Adult Health*

Figure A3. Predicted probability (standard errors) of suicide ideation by weight discrimination and gender—overweight and obese subsample (N=8,614)


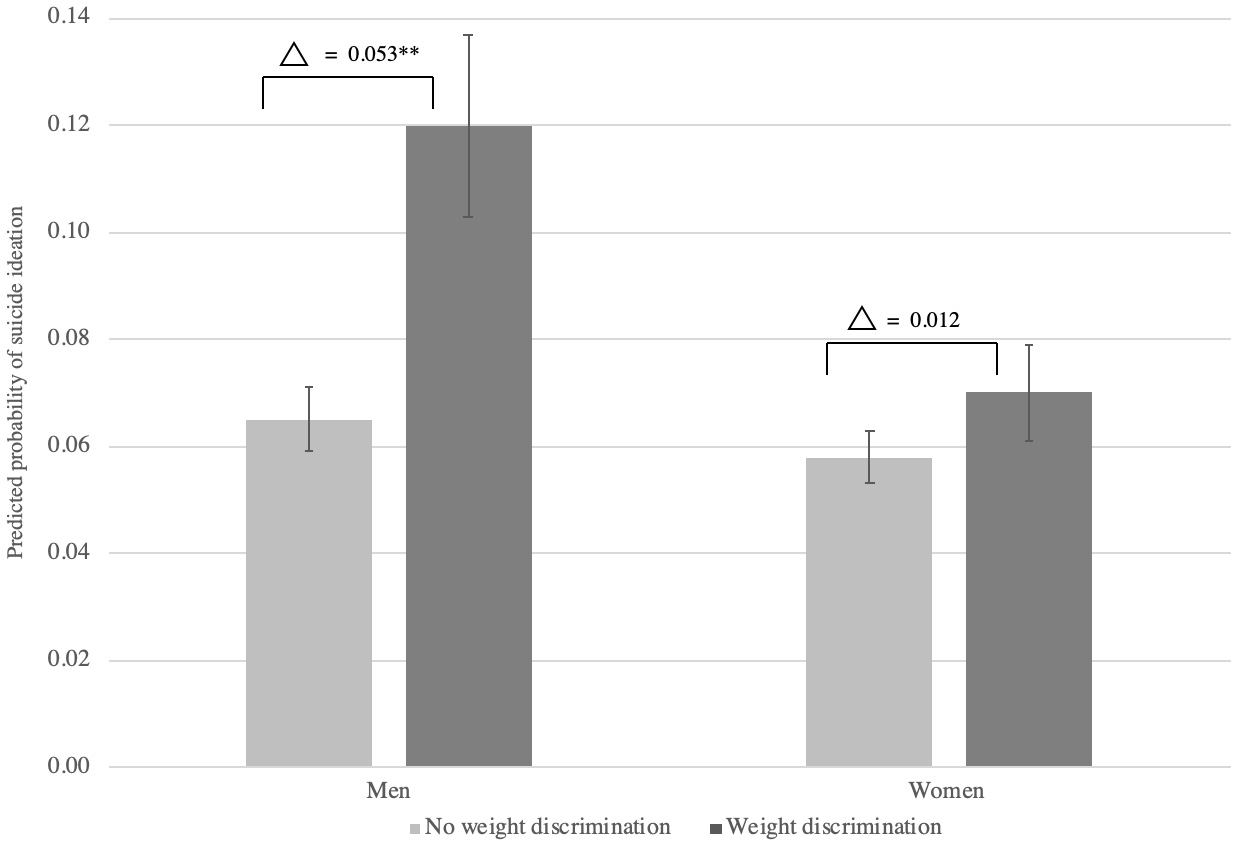


***p<0.001 ** p<0.01 *p<0.05

Δ = 0.048* between men, weight discrimination and women, weight discrimination

Δ = 0.007 between men, no weight discrimination and women, no weight discrimination

Second difference = 0.041*

*Source: National Longitudinal Study of Adolescent to Adult Health*

Figure A4. Predicted probability (standard errors) of suicide attempts by weight discrimination and gender—overweight and obese subsample (N=8,614)


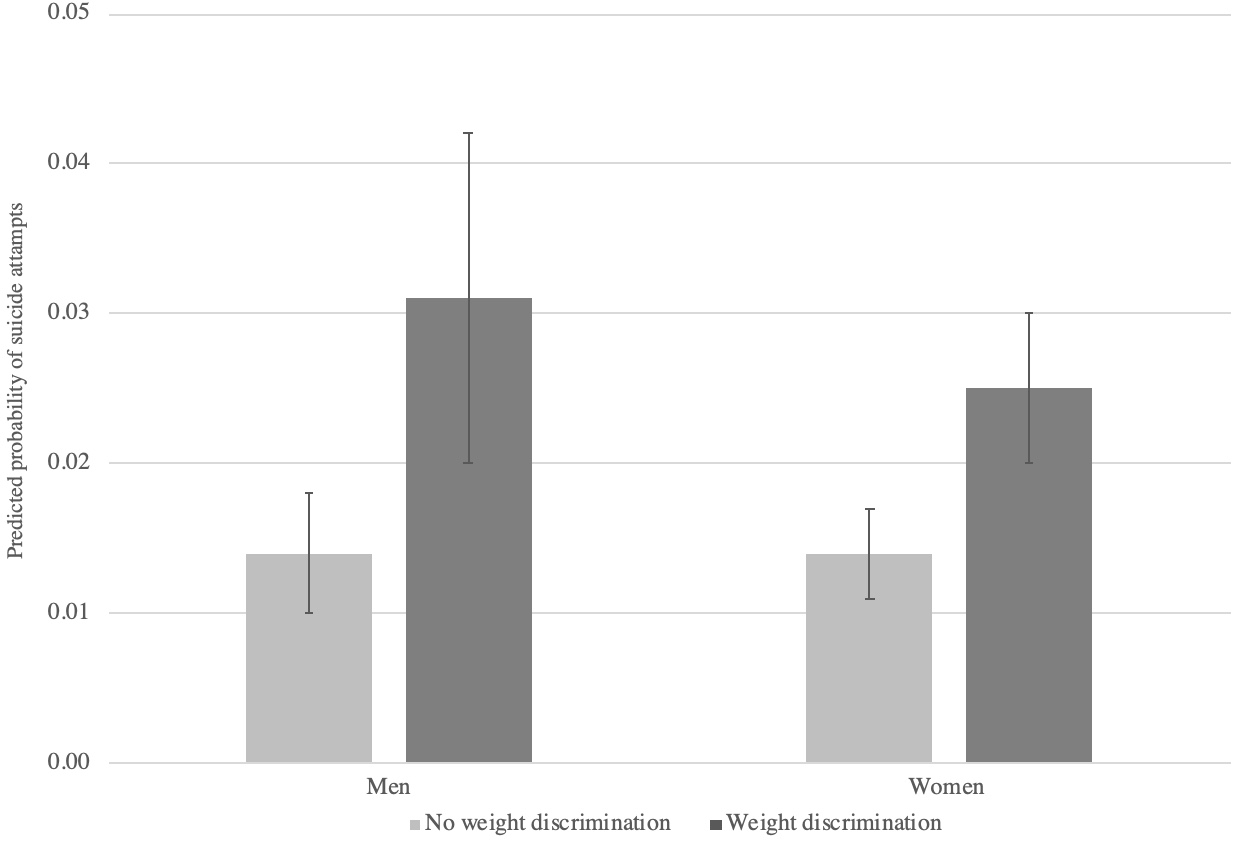


Note: there are no significant differences (Δ) at p<0.05 level between any of the categories

*Source: National Longitudinal Study of Adolescent to Adult Health*
